# Seasonal variability, long-term distribution (2001–2014), and risk assessment of polar organic micropollutants in the Baltic Sea

**DOI:** 10.1007/s11356-021-13254-5

**Published:** 2021-03-23

**Authors:** Kathrin Fisch, Berit Brockmeyer, Wolfgang Gerwinski, Detlef E. Schulz-Bull, Norbert Theobald

**Affiliations:** 1 Leibniz-Institute for Baltic Sea Research, Warnemünde, Seestr. 15, 18119 Rostock, Germany; 2 Federal Maritime and Hydrographic Agency, Bernhard-Nocht Str. 78, 20359 Hamburg, Germany

**Keywords:** Herbicides, Perfluorinated compounds, Pharmaceuticals, Polar micropollutants, Baltic Sea, Long-term trend. Risk assessment

## Abstract

From 2001 to 2014, 13 surveys were conducted in the Baltic Sea, to determine its pollution of 50 micropollutants. The investigations focused mostly on the German western Baltic Sea; in 2008, one survey covered the entire Baltic Sea. Various groups of herbicides (such as triazines, phenoxyacetic acid, phenylurea), perfluoroalkyl substances, pharmaceuticals, and industrial products were analyzed during these surveys. The highest concentrations (median 1 to 4 ng/L) were observed for atrazine, simazine, chloridazone, 2,4-dichlorophenoxyacetic acid, benzotriazole, primidone, and carbamazepine. Most micropollutants exhibited a relatively homogenous spatial distribution, though some herbicides show elevated concentrations in certain regions (e.g., Odra estuary), indicating a riverine input. The data set was analyzed, both for seasonal influences and long-time trends. Some herbicides exhibited higher concentrations during summertime. Both upward- and downward-directed time trends could be identified for some herbicides and perfluorinated compounds. For most of the detected compounds, a low-risk quotient was calculated. Only the occurrence of carbendazim could potentially pose a higher risk to the Baltic Sea.

**Supplementary Information:**

The online version contains supplementary material available at 10.1007/s11356-021-13254-5.

## Introduction

While long-lasting monitoring programs quite well-document information about the burden of the marine environment by classical non-polar pollutants such as chlorinated hydrocarbons (CHs) or polycyclic aromatic hydrocarbons (PAHs), the knowledge of the contamination by polar organic micropollutants is by far less described and evaluated (HELCOM 2010; Theobald 2011; Abraham et al. 2017; HELCOM 2018; Lang et al. 2018). The term micropollutant assembles many diverse compounds that are of anthropogenic origin in the environment. Compounds such as herbicides (e.g., triazines, phenoxyacetic acid, phenylurea, and miscellaneous), perfluoroalkyl substances (PFASs), pharmaceuticals and personal care products (PPCPs), and industrial products are regarded as polar micropollutants (Hollender et al. 2008; Loos et al. 2009; Nödler et al. 2014). Due to their polar character, most of them are water-soluble and can be detected in the aquatic phase and transported with the river water into the marine environment (Reemtsma and Jekel 2006; Loos et al. 2009). In terms of PFASs, as they are volatile, they can also be transported through the atmosphere into the marine environment (Prevedouros et al. 2006). For perfluorooctane sulfonic acids (PFOSs), the riverine input into the sea is more important source than the atmospheric deposition, e.g., Baltic Proper 172 kg/year atmospheric deposition versus 602 kg/year estuarine export (Lindim et al. 2016). Whereas for the perfluorooctanoic acid (PFOA), the atmospheric deposition is of greater relevance, e.g., Baltic Proper 689 kg/year atmospheric deposition versus 386 kg/year estuarine export (Lindim et al. 2016). As some of the PFOSs are known for their toxicity, bioaccumulation, and persistence in the environment, they have been regulated by the European Union, and were added to the Annex B list of “persistent organic pollutants” by the Stockholm Convention in 2009 (European Commission 2005a, b, c , 2006, 2007; Convention 2009). Many other polar micropollutants are not regulated yet.

This study aimed to present, analyze, and evaluate long-term results of seawater analysis obtained by the Federal Maritime and Hydrographic Agency (BSH) and the Leibniz Institute for Baltic Sea Research (IOW) during the last decade (2001–2014) to identify spatial hot spots as well as time trends of 50 polar organic micropollutants in the Baltic Sea.

## Material and methods

### Chemicals

Calibration standards for pesticides were purchased from Dr. Ehrenstorfer/LGC as neat materials, single solutions, or mixtures. Perfluoroalkyl substances were delivered from Wellington Laboratories/Campro as single solutions or mixtures. Labeled compounds (Deuterium and 13C) were used as internal standards. Methanol was used for standard solutions, SPE elution, and HPLC mobile phase (MeOH HPLC-analyzed, Baker). Ammonium acetate (p.a. Merck) and acetic acid (p.a. 96% Merck) were used for buffer solutions. Pure water for HPLC separations was prepared by a pure water system (Milli-Q academic A10, Millipore) until 2007, followed by bottled water (HPLC water, Baker). HPLC/MS-spectrometer was operated with Nitrogen Gas 5.0 (Air Liquide). For further information, see Tab. S1.

### Sampling station

**Fig. 1:**
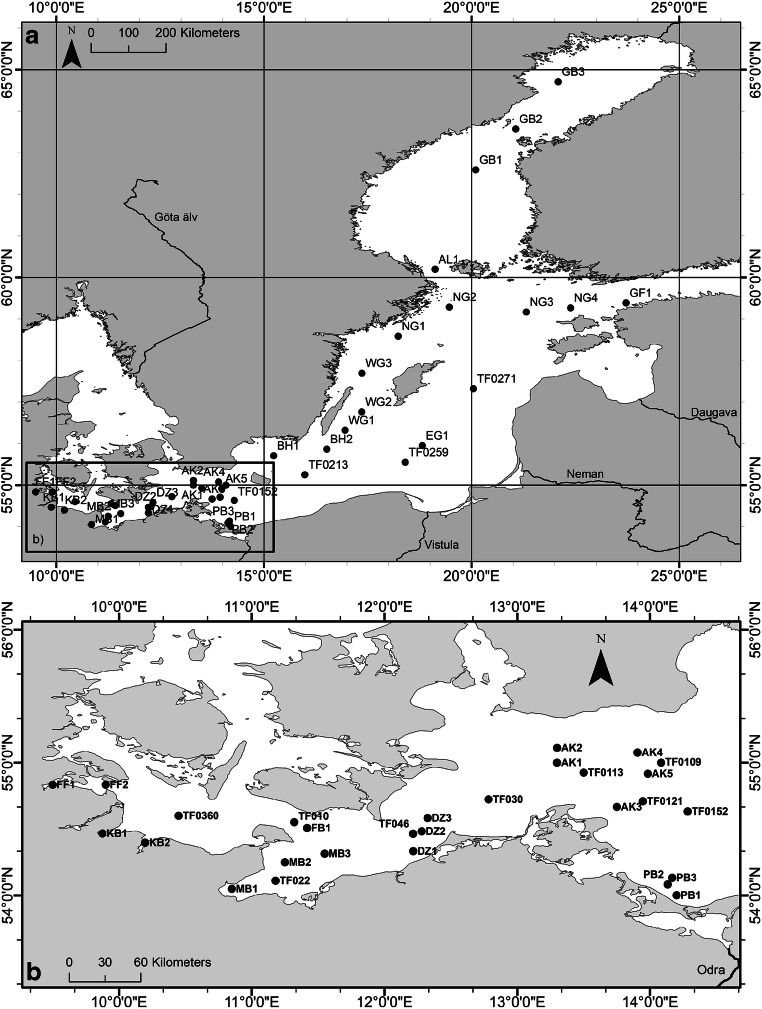
Sampling locations of all surveys from 2001 to 2014. **a** All sampling stations in the Baltic Sea. **b** Excerpt of sampling map southwestern Baltic Sea. More details about the station data can be found in Tab. S3

Most of the sampling was done during routine monitoring of the Leibniz Institute for Baltic Sea Research Warnemünde (IOW) from 2009 to 2014 at 7 (until 2009) to 9 (from 2010) stations in the western Baltic Sea (Fig. 1a, b). A similar station net was sampled from 2001 to 2005 by the Federal Maritime and Hydrographic Agency of Germany (BSH). In addition, the central and eastern part of the Baltic Sea was sampled on a research cruise during 2008 by R/V Maria S. Merian. The cruises from 2001 to 2008 were done in summer (June to August), while later samplings occurred during wintertime (January and February). Sampling details are presented in the supplements in Tab. S2 and S3. During all campaigns, a total of 133 water samples were taken and analyzed for 50 micropollutants. Some results of the perfluoroalkyl substances from the cruises GA442 and MM03/08 have been published in Theobald et al. (2007) and Kirchgeorg et al. (2010), respectively (Tab. S15).

### Water sampling and solid phase extraction

Samples from 5 m below surface (2001–2008) were collected in 10 L glass bottle samplers, and the internal pump system of the ship’s inlet was used from 2009 to collect samples at 4 m depth. An internal standard solution was added to each sample prior to further treatment for quantification calculations.

Solid phase extraction (SPE) was applied to enrich micropollutants for the analysis by high-performance liquid chromatography coupled with a tandem mass spectrometer (HPLC-MS/MS). Sample volumes of 6 to 9 L were pumped through 12 mL SPE columns containing 1.7 g polymer adsorber (samples from 2001 to 2009); 2.1 L samples were applied since 2009. To avoid clogging of the adsorber column, a 12 mL column filled with 1 g of glass wool was connected prior to flow direction. SPE adsorber material was Chromabond HR-P® (Macherey & Nagel, Düren, Germany) for all samples, except Strata-X® (Phenomenex, Aschaffenburg, Germany) for the AL430 cruise. Loaded SPE columns were eluted with methanol buffered with 5 mM ammonium acetate and 2.5 mM acetic acid. The solvent was vaporized to a final extract volume of 0.5 mL. The reduced sample volume since 2009 was an adoption to the higher sensitivity of the new MS/MS spectrometer. Changes in sampling and measurement features were checked by internal and external quality assurance or regular inter-comparison tests (Tab. S5).

### HPLC-MS/MS analysis

All samples were analyzed by HPLC-MS/MS, but the device configuration was subject to changes during the investigations. From 2001 to 2009, an MS/MS API 2000 (AB Sciex, Darmstadt, Germany) was used and then replaced by the Model 5500 QTrap of the same brand. Both systems were operated with a turbo ion spray probe. An HPLC Agilent Series 1100 was used for chromatographic separation from 2001 to 2012, which was then replaced by an HPLC Ultimate 3000 Series (Dionex/Thermo Fisher Scientific, Idstein, Germany). A combination of two HPLC columns (Synergi Polar RP, 4 μm particle size, 50 × 2 mm, and Synergi Hydro RP, 4 μm particle size, 75 × 2 mm, Phenomenex, Aschaffenburg, Germany, respectively), with a security guard column (Aqua C18, 4 × 2 mm, Phenomenex, Aschaffenburg, Germany), was used for separation with the Agilent HPLC system. The Dionex system was operated with a Kinetex C18 column (2.6 μm particle size, dimension 100 × 2 mm, Phenomenex, Aschaffenburg, Germany). The mobile phases were water (A) and methanol (B), each containing ammonium acetate and acetic acid. The concentrations of ammonium acetate and acetic acid were 10 mM each for the API 2000 spectrometer and 5 mM each for the 5500QTrap system. Gradient programs were carried out for elution and separation. The gradient program started at 15% B was increased up to 95% B with 220 to 300 μL/min as flow rates.

Ionization was carried out in positive and negative electrospray ionization mode, and the mass analysis was performed by scheduled multiple reaction monitoring. Ionization and mass transition parameters of LC-MS/MS analysis are listed in Tab. S4. Certified calibration standards were adopted from the routine mass spectrometer analyses of the BSH monitoring program. The calibration ranges of target analytes ranged from 0 to 10 ng/mL with average internal standard concentrations of 5 ng/mL. Analyte concentrations were calculated based on the relation of the analyte peak area to the internal standard peak area, in units of ng/mL extract. Extract concentrations were corrected by the arithmetic mean of field blank data. Concentrations were controlled for the limit of quantification (LOQ) and limit of detection (LOD). Finally, the absolute concentration in the sample extract (ng/mL) was converted into concentrations of target analytes in units of ng/L of the water sample. Achieved LOQ, recovery rates, and quality assurance are summarized in Tab. S5. As the new MS/MS spectrometer had better sensitivity and selectivity, the target list of micropollutants could be expanded since 2009.

The graphic artwork was created with RStudio (Fig. 3), ArcMap® 10.7.1 (Figs. 1, 4, 6, 5), and SigmaPlot 13.0 (Fig. 2, 7, 8, 9).

**Fig. 2:**
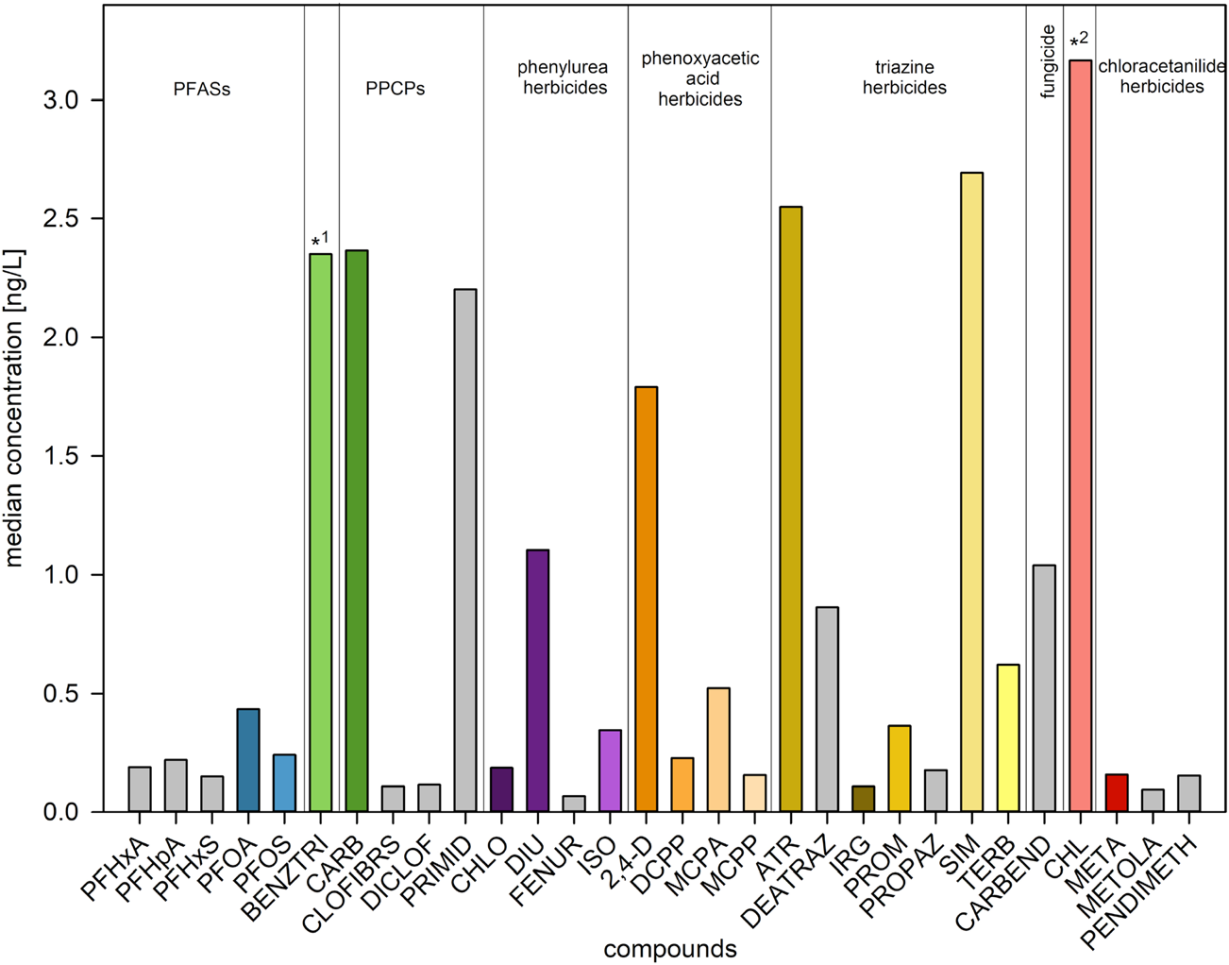
Median concentrations (2001–2014) of the most frequently detected compounds of the total data set. Compounds displayed in color will be further discussed. *^1^ corrosion inhibitor, *^2^ pyridazinone herbicide; Data: see Tab. S6, S7

## Results and discussion

### Most frequently detected compounds in the Baltic Sea

In this study, 50 different organic micropollutants of mid polarity from the following application and chemical classes were analyzed: 2 industrial and household chemicals, 9 perfluoroalkyl carboxylic acids (PFCAs), 7 PPCPs, and 32 herbicides covering the classes phenylureas (6), phenoxycarboxilic acids (4), triazines (9), and others (13) (Tab. S1). The observations of the German routine monitoring program performed in the North were the base for the selected micropollutants. The median concentration of the most detected compounds in all 133 samples ranged from *<* LOD to 3.2 ng/L, and are displayed in Fig. 2. In Tab. S6, more statistical details of all measured compounds are summarized. Compounds that were measured in small concentrations (median *<* 0.15 ng/L) or in few samples (Fig. 2, displayed in gray) were excluded from further discussion (see Tab. S6). As metoprolol was found below its LOQ and was only analyzed since 2013, it is also excluded from further description. The compounds carbendazim (CARBEND), benzotriazole (BENZTRI), and PFASs were analyzed since 2009 (Tab. S12). Compounds of each class, measured with the highest median concentrations are further discussed in this paper (Fig. 2, displayed in color).

Among the herbicides, chloridazon (CHL) showed the highest median concentration (3.2 ng/L) followed by simazine (SIM) and atrazine (ATR) (2.7 ng/L and 2.5 ng/L, respectively), 2,4-dichlorophenoxyacetic acid (2,4-D), diuron (DIU), terbuthylazine (TERB), MCPA, and isoproturon (ISO) (0.3 to 1.8 ng/L). From the PFASs, perfluorooctanoic acid (PFOA) and perfluoroctylsulfonic acid (PFOS) exhibited the highest values (0.4 ng/L and 0.2 ng/L, respectively). The pharmaceuticals carbamazepine (CARB) and primidone (PRIMID) were detected with concentrations of 2.4 ng/L and 2.2 ng/L respectively, as well as the complex-forming agent BENZTRI (2.3 ng/L). Many of the compounds (Fig. 2) showed low variation coefficients of 15 to 100%, which is an indication for a low variance in space and time. However, compounds of low concentrations exhibit higher variabilities of up to 393% (Tab. S6).

### Spatial distribution and input sources in the western part of the Baltic Sea

The main survey area is the German exclusive economic zone (EEZ) in the western Baltic Sea, where 12 surveys from 2001 to 2014 were carried out (Tab. S2, S3). In addition, one survey, covering the whole Baltic Sea area up to the Bothnian Sea and the Gulf of Finland, was done in summer 2008 (Tab. S2, S3).

**Fig. 3:**
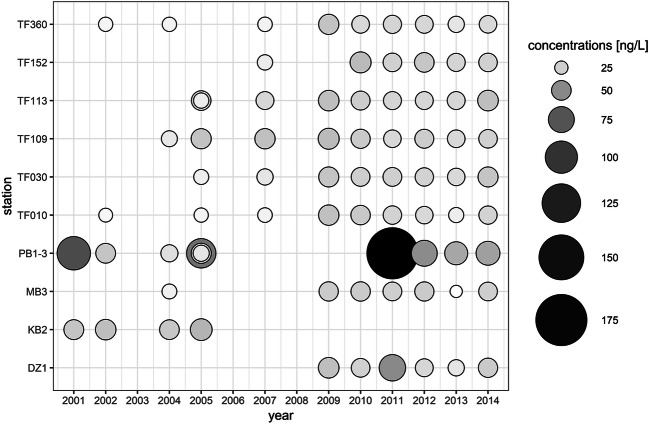
Spatial distribution of the total sum of all detected micropollutants at most frequently measured stations from 2001 to 2014. No sampling was carried out in the years 2003, 2006, and 2008. In 2005, more than one sampling was done at the stations TF113 and PB1-3. Data: see Tab. S7

In order to get a first spatial and temporal overview of the distribution of the ten, most frequently sampled stations, the total concentrations were calculated for each year from 2001 to 2014 (Fig. 3, data Tab. S7). The different stations show similar total concentration medians over time, which was indicated from the low overall variation coefficients. When the sampling campaigns are subdivided into two periods (2001–2007 and 2009–2014), the general trend shows a slight increase in the median concentration by about 6 ng/L, from first to second period (2001–2007, 18.1 ng/L, *n* = 22; 2009–2014, 24.5 ng/L, *n* = 47; excluding station PB1-3). Furthermore, the detected total concentration range until 2007 (13.2–33.7 ng/L, 2001–2007) is smaller than the range from 2009 to 2014 (10.1–50.2 ng/L), reflecting the slight increased median concentration. The station which can easily be identified by its high concentrations is the station PB1-3, which is situated close to the Odra mouth (Fig. 1b). The concentrations are higher at this station than at any other sampled stations. As the Odra is the only significant freshwater input in this sampling area, these higher concentrations can be explained.

**Fig. 4:**
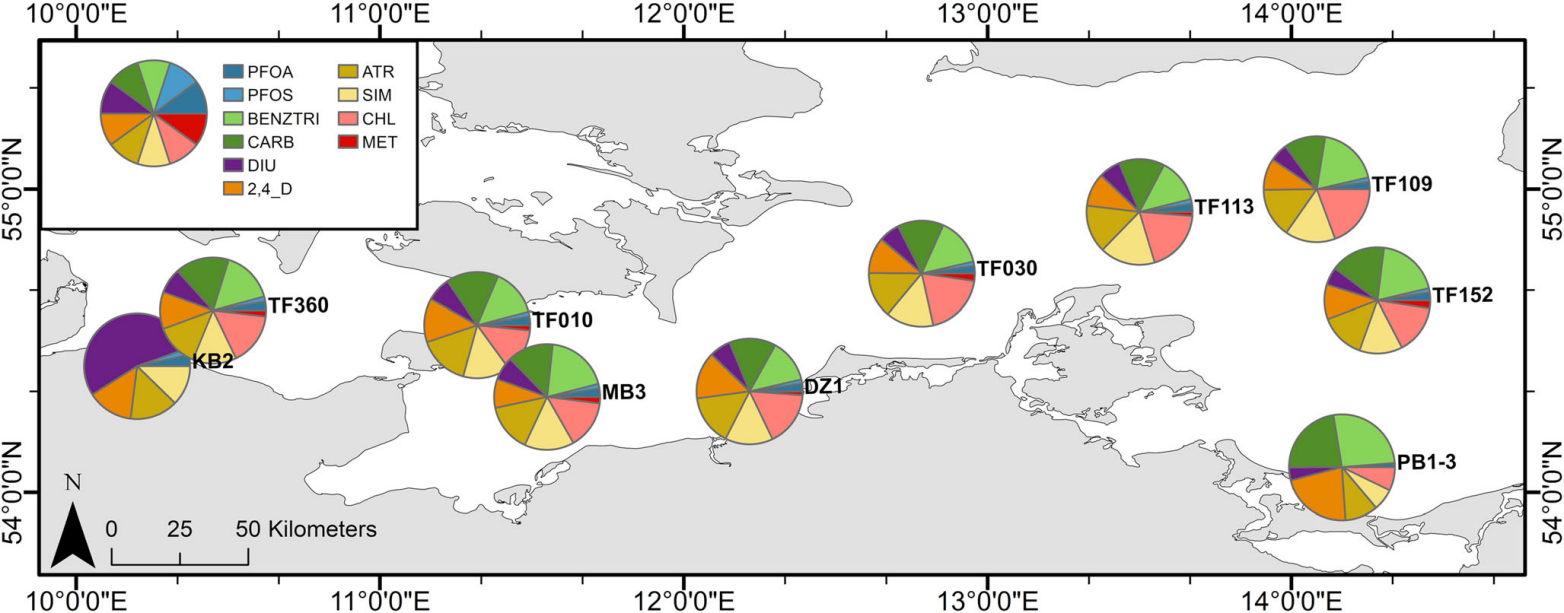
Composition (%) of the top 10 detected micropollutants at the most frequently sampled stations from 2001 to 2014. Data: see Tab. S7

The Odra near station PB1-3 not only shows elevated concentrations but also distinct differences in its compound composition (Fig. 4). The input is high for the complex-forming agent BENZTRI and most pharmaceuticals (CARB, Diclofenac (DCF), and PRIMID, Fig. 4, Tab. S1, S7). Furthermore, high concentrations were detected for the herbicides, ISO, 2,4-D, chlorotoluron (CHLO), MCPA, TERB, and less for DIU. Odra’s input at the station PB1-3 is in the low range of 0.002–4.5 ng/L for most PFASs, SIM, and CHL compared to other stations. A special case is station KB2, as it seems as though DIU was detected at much higher concentrations than at any other station (Fig. 4). However, KB2 was sampled only until 2005. Compounds like BENZTRI and CARB were not sampled until 2009 and thus were not measured at KB2. Despite that, the other stations display a similar composition pattern among each other, which indicates an evenly distribution of the compounds in the western Baltic Sea. Especially, ATR, 2,4-D, and CARB occur with low variability between the stations (excluding KB2, PB1-3).

**Fig. 5:**
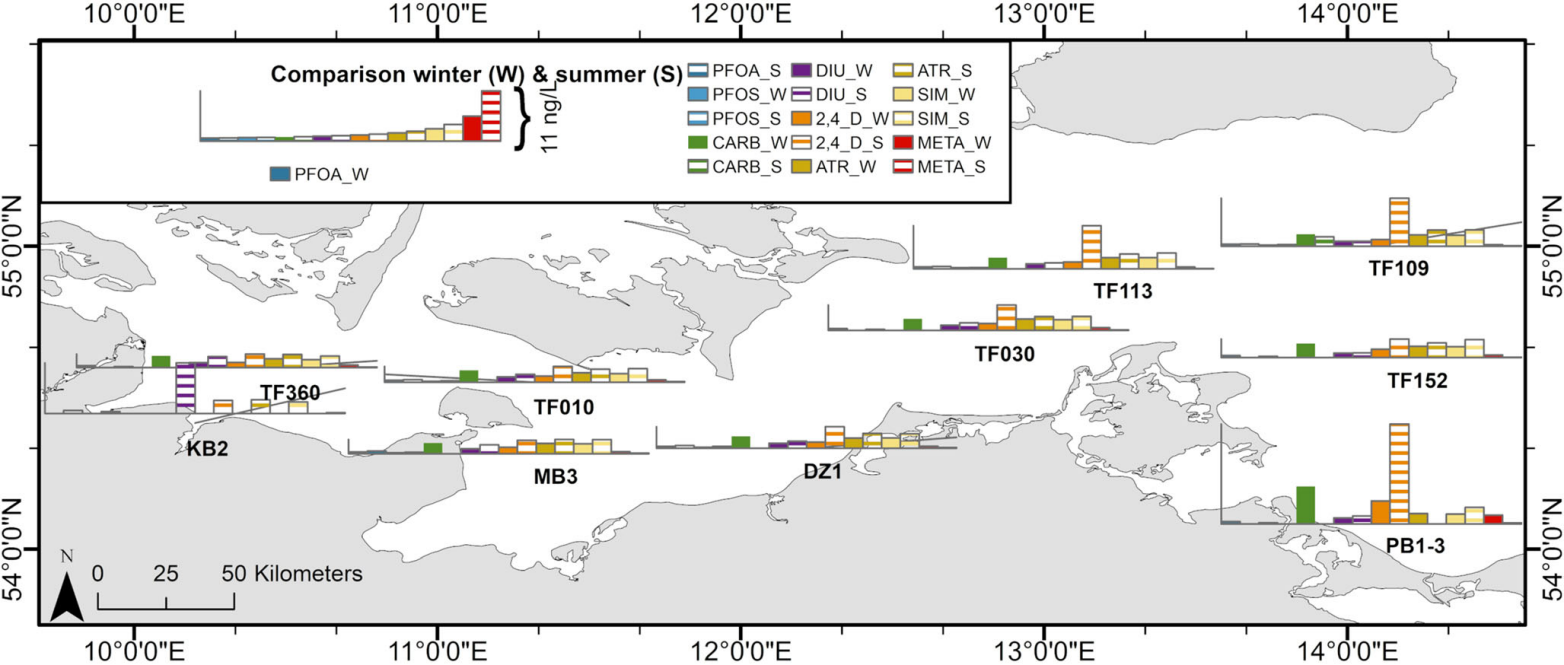
The median concentration of selected compounds for winter (W, full-colored column) and summer (S, stripped column) cruises. Data: see Tab. S8

By the detailed analysis of single cruises, it turns out that there are distinct spatial differences for some compounds between summer and winter surveys (Tab. S7, S8). The summer surveys were conducted, from 2001 to 2005 and the winter surveys from 2009 to 2014. Therefore, separate medians for summer and winter cruises were calculated, for the respective stations (Tab. S8). As can be seen in Fig. 5, during the winter cruises, the median concentrations for the most occurring compounds (PFOA, CARB, DIU, 2,4-D, SIM) are homogenous from the west (KB2) to the east (TF152); only at station PB1-3, elevated concentrations were detected (Tab. S8). Some compounds like ATR, SIM, PFOA, PFOS-1, and CARB show a homogenous distribution during the summer period as well (Fig. 5). The high summer concentrations for PFOA, PFOS-1, and ATR at PB1-3, shown in Tab. S8, should be interpreted with precaution as they are statistically of low relevance (only one cruise). However, for most herbicides, on average, high spatial variations are observed in summer. Especially, DIU and Irgarol (IRG) exhibit high hot spots at the western coastal stations (KB2, MB1, Tab. S8). Even though DIU is prohibited as a pesticide, in Germany since 2009, it is still used as a biocide and was detectable in the marine environment during the winter cruises (2009–2014) (European Commission 2007). Other herbicides (ISO, MCPA, TERB) show elevated concentrations at the western stations as well, but to a much smaller extent (Tab. S8). For TERB, it was reported that its occurrence is of high spatial and temporal variability (Orlikowska et al. 2015). Thus, for most herbicides, more or less pronounced concentration gradients can be observed during summer from west to east.

In contrast, 2,4-D shows high summer concentrations at the eastern stations, starting at DZ1 and TF030 (north of the Darss) and peaking at the Arkona Basin (TF113 and TF109). 2,4-D shows high concentrations at the Odra near station PB1-3. Another compound with large input from the Odra is CARB (PB1-3, 7.2–12.2 ng/L), despite that it does not show elevated concentrations at the Arkona Basin (TF109, 1.6–2.3 ng/L, Fig. 5, Tab. S7). In 2013, Björlenius et al. (2018) conducted widespread pharmaceutical screening in Baltic Sea coastal waters. The detected CARB concentrations during this study are in similar range (2.5–9.1 ng/L, 2.1–3.3 ng/L, respectively) as station PB1-3 and TF109. Furthermore, the study presented a similar decrease from the near coastal water in the Odra Bay towards the Arkona Basin. ISO and TERB show elevated concentrations at PB1-3 to a medium extent (Tab. S8). For most other compounds, the Odra input seems to be less pronounced.

### Spatial distribution in the central and eastern Baltic Sea

**Fig. 6:**
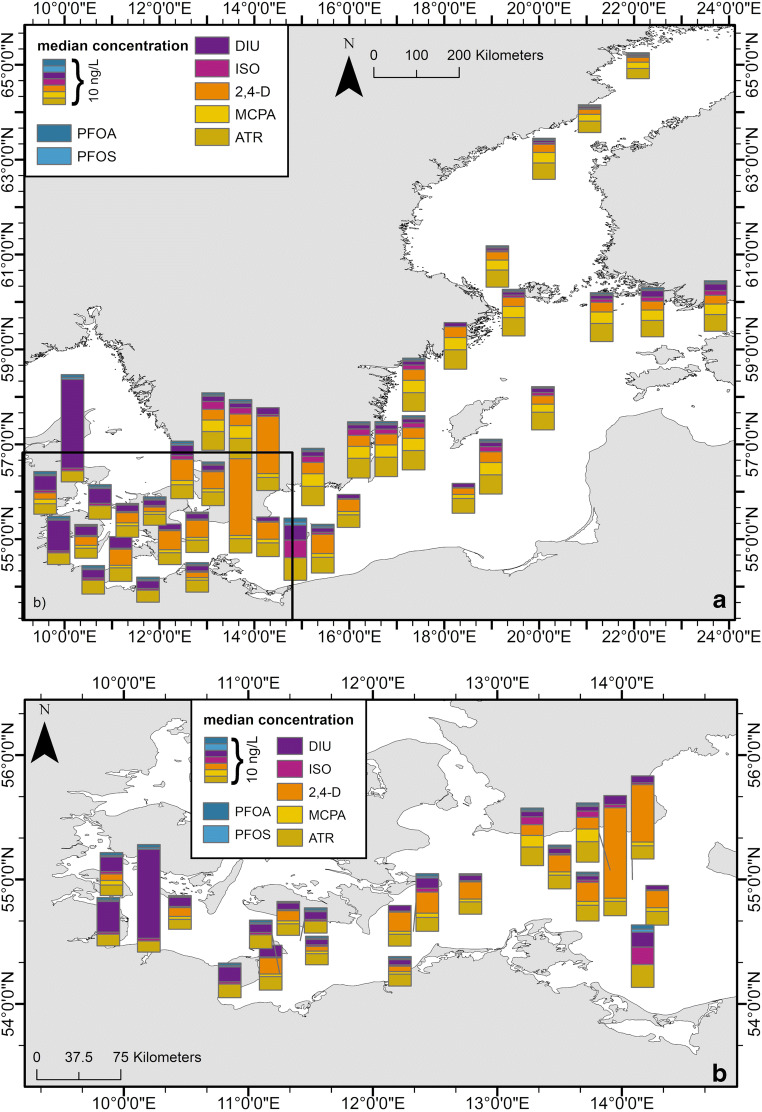
Median distribution of the most prominent micropollutants along longitudinal trends from 2005, 2007–2009 **a** Baltic Sea **b** close-up western Baltic Sea. Data: see Tab. S9

The station net, sampled during the MM0803 cruise, allowed the investigation of the occurrence of the determined compounds in the central and eastern part of the Baltic Sea (parts of the PFAS data has been published by Kirchgeorg et al. (2010)). The median results are displayed in Fig. 6a, b (data; Tab. S9). Most of the prominent compounds were detected in the east, at concentrations similar to the western and central part of the Baltic Sea (median east 10.7 ng/L (*n* = 15), median west 10.0 ng/L (*n* = 25), Tab. S9, east-west split at N 16° 30′ 0″). Thus, there is a fairly homogenous distribution of micropollutants in the Baltic Sea, although the concentration range in the west is higher than in the east (west 5.9–27.4 ng/L, east 5.9–12.9 ng/L, Tab. S9). At the eastern edge of the survey area, the concentrations split into lower concentrations at the northern stations of the Bothnian Sea (GB2 and GB3), and higher concentrations at the southern stations of the outer Gulf of Finland (GF4 and NG3-4) (Fig. 6a). The degree of this “splitting” is different for the various micropollutants. In the western part of the Baltic Sea, the three most dominating compounds are DIU, 2,4-D, and ATR, whereas, in the eastern part, ATR, MCPA, and 2,4-D are the more dominating compounds, but at a marginally lower level.

A homogenous distribution, with gradually declining concentrations from west to north-east, is observed for PFOA. No significant variability, for the Gulf of Finland and only a small decrease for the Bothnian Sea, was identified (Fig. 6a). The detected concentrations of PFASs, during the MM0308, are in a similar range as published PFAS concentrations by Kirchgeorg et al. (2010) for the same year (Tab. S15). Similar distributions are encountered for PFOS and ISO (Fig. 6a, b, Tab. S9). ISO was increased up to 4.0 ng/L only at station PB1-3 (general median: 0.5 ng/L). At this station, the concentration of ATR is also increased (5.2 ng/L, Fig. 6b) in comparison to the other stations in the Baltic Sea, where ATR was detected at homogeneous concentrations with low variation (median 3.2 ng/L). The concentrations of DIU and 2,4-D are marginally increased in the western part (median 1.5 ng/L, 2.8 ng/L, respectively) in comparison with the eastern part (median 0.9 ng/L, 2.0 ng/L, respectively). A strong increase for DIU was detected at station KB2 (20.2 ng/L) and KB1 (7.0 ng/L) in the western Baltic Sea (Fig. 6b, Tab. S9), which are close to the outlet of the Kiel Canal. The highest concentrations of 2,4-D were detected in the western Baltic Sea at station TF121 (20.6 ng/L) and TF109 (13.0 ng/L).

### Temporal Aspects

The general overview of the data set, depicting the median concentrations of the indicator contaminants of the 13 surveys from 2001 to 2014, is presented in Fig. 3. Additional statistical data is presented in the supplements (Tab. S10). For some compounds, the data indicates that overall both clear upward (e.g. PFHXA, Metazachlor) and downward (e.g., ATR, SIM) temporal trends are detectable. However, for many compounds, high variability is observed, which might indicate an influence of special local or seasonal effects making trend analysis difficult. The data set is divided into two sampling periods: from 2001 to 2008, sampling took place during summer (May to August, Fig. 5), and from 2009 to 2014, sampling occurred in January and February. In addition, the early cruises in summer contained some additional coastal stations, semi-enclosed bay areas, in the western part of the Baltic Sea. Unfortunately, there was no sampling in the winter and summer of the same year. Thus, there remains some uncertainty concerning the interpretation of the observed temporal effects.

The contaminants can be subdivided into three groups according to their temporal and spatial behavior (Fig. 5). One group exhibits a uniform behavior without great differences between summer and winter and no significant spatial differences. ATR, SIM, and PFASs belong to this group. They can be characterized by the absence of large local acute inputs. The temporal behavior of no seasonal variation for ATR, SIM, and PFASs has also been observed in other coastal waters (Carafa et al. 2007; Hu et al. 2010; Zhao et al. 2015).

A second group (DIU, IRG, and 2,4-D) is characterized by high variability in summer, with distinct local hot spots and high variability between single sampling campaigns (during summer sampling). DIU and IRG show high concentrations in the most western part of the Baltic Sea (Figs. 4, 5). Remarkably, 2,4-D shows elevated concentrations in an area north of Rügen and the Arkona basin (TF030, TF113, and TF109). It is difficult to decide on trends for these compounds, due to their high and variable concentrations, in the early years of the monitoring period. The observable downward “trend” can be caused by the seasonal effect. Furthermore, Kot-Wasik et al. (2004) observed higher phenoxyactic herbicide concentrations during springtime in the Gulf of Gdansk (Baltic Sea) as well.

In a third group, compounds can be summarized without any significant high local elevations in the summer but with slightly elevated concentrations during summer. Trends are often detected for these compounds. In the case of decreasing temporal trends, it remains more or less a great uncertainty. In the case of upward trends, like TERB and METOLA, they can be accepted as reliable (Tab. S10, S11). The seasonal variation, with elevated concentrations during the spring/summer time, for TERB and METOLA has been observed in the Sacca di Goro lagoon (Italy) as well (Carafa et al. 2007).

#### Long-time trends

**Fig. 7:**
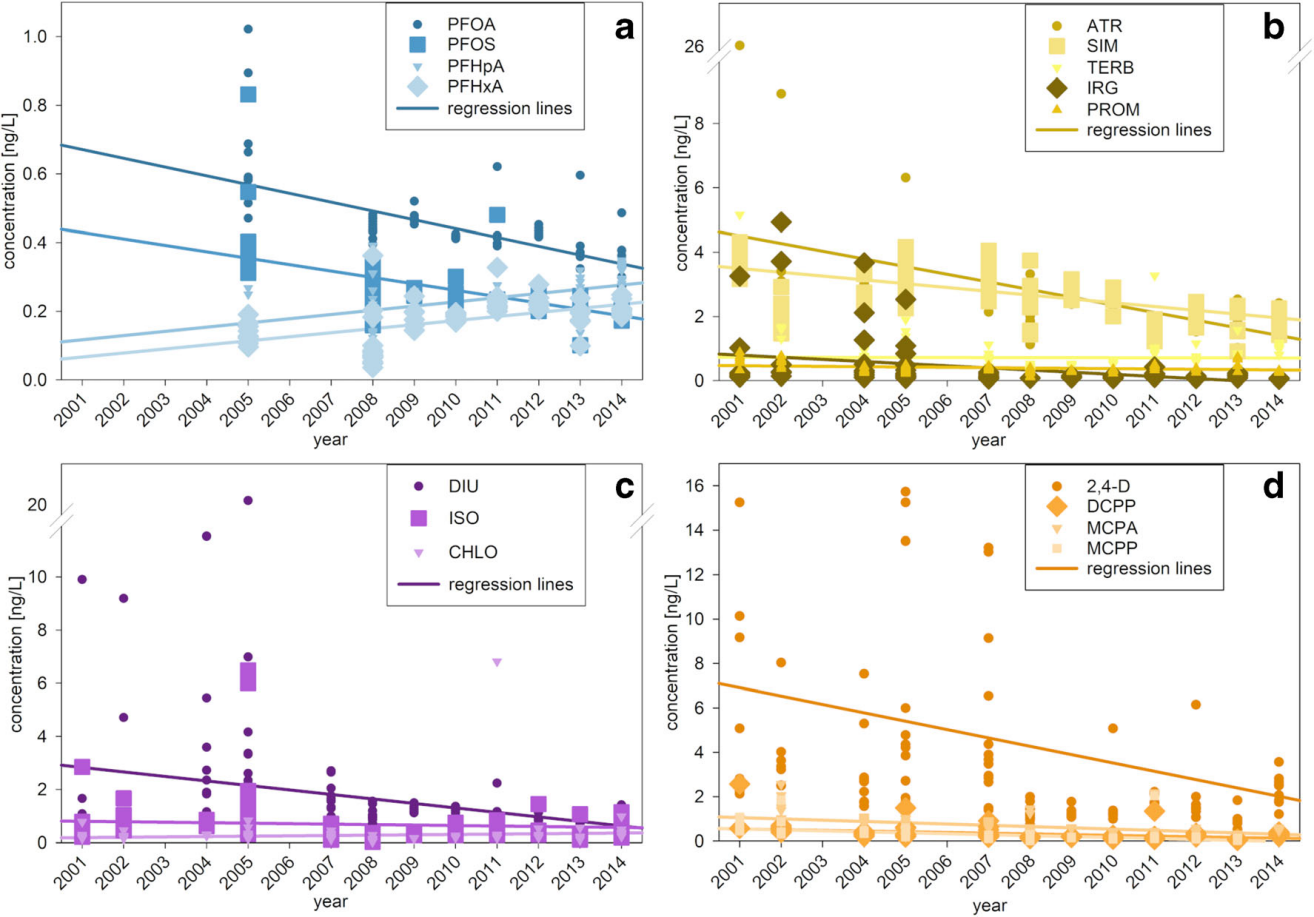
Time trend of selected mircopollutants in the Baltic Sea from 2001 to 2014. **a** Perfluoroalkyl substances. **b** Triazine herbicides. **c** Urea herbicides. **d** Phenoxyherbicides. Data: see Tab. S11, S12

Based on the above-described sensitivity towards possible seasonal effects (summer/winter time), the time courses of the compounds were analyzed for selected areas (from west to east): TF360, TF010 and MB3, DZ1 and TF030, and TF113 and TF109 (pairing of stations was selected based on the similarity of the stations’ data and due to statistical representativeness). In Tab. S11, the results of the trend analysis, as well as the sensitivity for seasonal effects, are summarized. The trends were identified by critical visual inspection of the graphically displayed data (Fig. 7). The presented calculated linear regression lines support the visual inspection but were not used for quantitative evaluations.

The class of PFASs shows no seasonal influence (group 1, sec. 3.4), therefore, trend analyses is quite reliable although the time period is shorter (2005 to 2015) (Fig. 7a, Tab. S11). The main compounds PFOA and PFOS showed a clear downward trend at all stations during the entire period. In contrast, the shorter chain C6- and C7-compounds PFHxA and PFHpA exhibit slightly increasing trends. Evidently, the voluntary abandonment of the C8 technology in 2002, by the main producer (3M), shows positive results with decreasing concentrations in the Baltic Sea. The PFAS concentration range over time is in a similar range as former conducted studies in the Baltic Sea (Theobald et al. 2007; Rostkowski et al. 2009; Ahrens et al. 2010; Kirchgeorg et al. 2010; Theobald 2011) (Tab. S15).

The group of herbicides exhibits more complex behavior. The triazines ATR and SIM are not influenced by seasonal effects (group 1, sec. 3.4) and show clear downward trends from 2001 to 2014 at all stations (26.0–1.0 ng/L, 4.3–0.9 ng/L, respectively) (Fig. 7b). These findings fit well with investigations of the literature and demonstrate positive effects of the ban in the mid 1990 (European Commission 2005c, a; Mai et al. 2013). In the 1990, levels of ATR near the coast and SIM in the south-western Baltic Sea reached up to 20 ng/L and 30 ng/L, respectively (Bester and Hühnerfuss 1993; Graeve and Wodarg 1996). Further offshore, they were detected at lower levels of 1.8–5.1 ng/L and 2.4–6.1 ng/L, respectively (Pempkowiak et al. 2000). The mean long-time trend (2001–2014) values (2.9 ng/L, 2.7 ng/L, respectively) are consistent with the study of (Pempkowiak et al. 2000). Moreover, the mean values of ATR and SIM (2.2 ng/L, 2.0 ng/L, respectively) of 2014 are consistent with the measurements done by Orlikowska et al. (2015) in the southern Baltic Sea (1.9 *±* 0.3 ng/L, 2.3 *±* 0.4 ng/L, respectively). ATR and SIM are listed as priority substances in the European Water Framework Directive (environmental quality standards 0.6 μg/L, 1.0 μg/L, respectively) (Union 2013). All detected concentrations during the long-time series did not exceed these environmental quality standards. PROM does not show a seasonal variability as well (Tab. S8), but at most stations, no time trend could be identified (Fig. 7b, Tab. S11). For TERB, a slight seasonal influence cannot be excluded, but slight upward trends can be observed at most stations. TERB is possibly replacing ATR and SIM, although its concentrations are much lower. Yet, its concentration range (0.2–5.2 ng/L) is in agreement with literature values (not detected–11 ng/L) (Bester and Hühnerfuss 1993; Pempkowiak et al. 2000; Nödler et al. 2013; Orlikowska et al. 2015). IRG shows a slight downward trend at the western stations TF010 to TF030, but merely any in the Arkona Basin (TF113 & TF109) and is generally decreasing (Fig. 7b, Tab. S11). High summer concentrations have been observed at the most western stations KB2 and MB3. Unfortunately, only data of concentration in summer between 2001 and 2005 are available, which do not allow a trend detection. However, all IRG concentrations are below the maximum concentration of 16 ng/L listed by the Water Framework Directive since 2013 (Union 2013). Furthermore, IRG has decreased since 1997, where it was detected in the Baltic Sea in a much higher concentration range (90–440 ng/L) (Biselli et al. 2000). The downward trend of IRG in the Baltic Sea coincides with the results of Orlikowska et al. (2015) who did not detect IRG in the Baltic Sea in 2014. The same was observed for the phenylurea DIU (Fig. 7c, Tab. S11). Due to its elevated concentrations in summer and local input sources, no general trends can be identified. Generally, slightly elevated concentrations for ISO were observed in summer, which makes the evaluation less reliable. Both concentrations of DIU and ISO are lower than the reported median concentration of Nödler et al. (2014) in 2009. All observed concentrations did not exceed the environmental quality standard of 0.2 μg/L and 0.3 μg/L, respectively (Union 2013). The concentration of CHLO is uniform at all stations showing no trends (Fig. 7c, Tab. S11).

The phenoxyacetic acid 2,4-D has a high variability at some local hot spots and is probably influenced by summer inputs (Fig. 7d, Tab. S11). Therefore, no reliable trends can be assigned, except for a decreasing tendency at the western stations. Similarly, DCPP and MCPA show a tendency to slightly decreasing trends, which are interfered by seasonal but not local effects (Fig. 7d, Tab. S11). The concentration of MCPP is below the reported median concentration (7.8 ng/L) by Nödler et al. (2014) in 2009.

METOLA and META show a clear upward trend at all stations (Tab. S11). Although CHL was detected at high concentrations, no trend can be identified, because of its high variability and short sampling period (since 2005 only) (Tab. S11). Also, trend evaluations of the pharmaceuticals and the complex former BENZTRI are limited by the short monitoring period (since 2008) (Tab. S11). For CARB, an upward trend seems to be indicated.

As was shown in the “Spatial distribution” section, the concentrations are homogenously distributed throughout the Baltic Sea, except for the semi-enclosed most western stations (KB2, MB1), and at the Odra mouth (PB1-3). No spatial trends could be identified for KB2 and MB1, because of the short sampling period (2001–2005) and the high variability. Also, PB1-3 did not show any trends because of the high variability of the data. For ATR, PFOA and PFOS elevated values have been observed at PB1-3, only at the beginning of the investigations (2001 and 2002 for ATR; 2005 for the PFASs) (Fig. 7a, b). In later years, these elevated values were not observed any longer. Thus, a significant decrease in input concentrations, by the Odra, might be assumed for these compounds.

### Comparison with the North Sea and German Bight

**Fig. 8:**
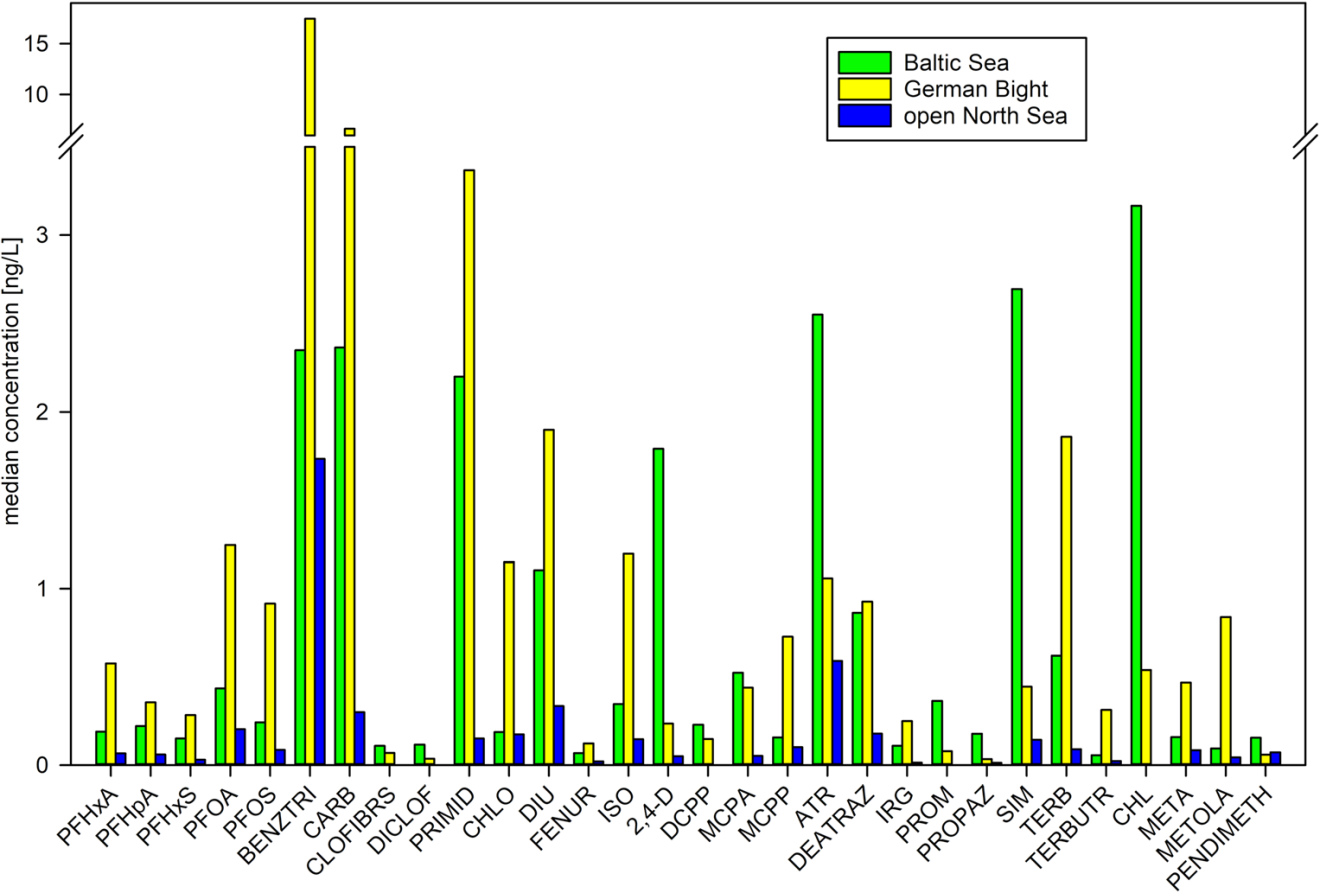
Comparison of median pollutant concentration of the Baltic Sea with those of the German Bight (GB) at coastal stations (S < 32) and the open sea region (S > 34). Data: see Tab. S13

Levels of polar organic micropollutants of the Baltic Sea were related to the monitoring data of the German Bight (North Sea) (Loewe 2009; Theobald et al. 2011; Loewe et al. 2013). Median micropollutant concentrations of the Baltic Sea were compared to median concentrations detected in coastal waters of the German Bight (salinity (S) *<* 32), the outer German Bight, and the central North Sea (with S *>* 34) (Fig. 8, Tab. S13). It was observed that concentrations of most contaminants were higher in the coastal waters of the German Bight than in the Baltic Sea. The ratio between the coastal German Bight and the Baltic Sea ranges from 0.2 to 14.7, indicating that the contaminant pattern considerably differs between these two regions (Tab. S13). In comparison to the open North Sea (S *>* 34), micropollutant concentrations in the Baltic Sea were 1.4 and 35.1 times higher (at max. 80.5 times higher), hence, demonstrating that the Baltic Sea is far from reaching “clean” open seawater state (Tab. S13). The complex former BENZTRI and the pharmaceutical CARB are the most dominant anthropogenic compounds in the coastal area of the German Bight and are introduced by the rivers Elbe and Rhine (Loewe 2009; Theobald et al. 2011; Loewe et al. 2013). Their concentrations are high in the Baltic Sea as well, where the major input is the Odra.

The PFASs show similar patterns in the North and Baltic Sea. However, in coastal areas of the German Bight, the median concentrations are about 2 to 3 times higher (Fig. 8). This circumstance was also observed by Ahrens et al. (2009, 2010). They reported that higher sums of PFASs were detected at German near-coastal stations than in the Baltic Sea or the open North Sea. Importance of this is that PFBS, a replacement compound for PFOA and PFOS, is even 12.3 times higher in the German Bight (Tab. S13).

Among the triazine herbicides, there are distinct differences in the individual compound patterns. ATR and SIM concentrations are significantly higher in the Baltic Sea than the German Bight (2.4 and 6.1 times higher, respectively) (Fig. 8). These are clearly old burdens, which are slowly washed out from the semi-enclosed maritime area of the Baltic Sea. However, even in the open North Sea, these compounds still show relatively high concentrations (max. 1.1 ng/L and max. 0.5 ng/L, respectively, Tab. S13), demonstrating their high persistence in the environment. Among the still licensed triazines, TERB is the dominating herbicide in the German Bight (1.9 ng/L), whereas it is much lower (0.6 ng/L) in the Baltic Sea (Fig. 8, Tab. S13). On the other side, the concentration of PROM is higher in the Baltic Sea (0.4 ng/L) than in the German Bight (0.08 ng/L). Among the phenylurea herbicides, DIU shows the highest concentrations in both seas (German Bight 1.9 ng/L, Baltic Sea 1.1 ng/L) followed by ISO and CHLO (Fig. 8, Tab. S13). In the group of phenoxyacetic acid herbicides, significant differences are shown. 2,4-D is the dominant herbicide (1.8 ng/L) in the Baltic Sea, but concentrations are low in the German Bight (0.2 ng/L) (Fig. 8, Tab. S13). In contrast, MCPP concentrations are much higher in the German Bight than in the Baltic Sea (0.7 ng/L and 0.15 ng/L, respectively). MCPA and DCPP occurrences are similar in both seas (Tab. S13). Among the other herbicides, CHL shows remarkably higher levels in the Baltic Sea, with a median concentration of 3.2 ng/L compared to 0.5 ng/L in the German Bight (Fig. 8, Tab. S13). For all other herbicides, higher concentrations were observed in the German Bight (Tab. S13).

Though the high ATR and SIM concentrations can be explained, by an old burden and the limited water exchange of the Baltic Sea, the reasons for the other observed differences in the compound patterns are less distinct. Differences can arise by the different main sampling periods as the Baltic Sea was mainly monitored in winter, whereas the German Bight was sampled during summer (Loewe 2009; Theobald et al. 2011; Loewe et al. 2013). However, differences in herbicide application can be a cause as well, due to historic, economic, or agricultural (crop cultivation) reasons. The lower general concentrations can be explained by the lack of large river input, as in the case of the German Bight (e.g., Elbe, Rhine). Because of this, the much lower concentrations of BENZTRI and CARB can be explained. Both are discharged by the Odra, but the main freshwater stream is directed to the east and does not influence most of the monitoring stations. For the PFASs, the Odra is no significant input source. As shown in the “Long-time trends” section, some herbicides in fact show seasonal dependence and higher concentrations during the summer, e.g., DIU and 2,4-D. This can explain the lower values because of leveling effect, due to their application periods, of the winter sampling campaigns. Although, this does not explain the observed different patterns: high 2,4-D and CHL, but low TERB.

### Ecological evaluation and risk assessment

Even though the observed concentrations of the determined micropollutants might appear to be relatively low, they do increase the pollution of the Baltic Sea. As there are not yet reliable eco-toxicological data available for most of the investigated compounds, it is difficult to assess the ecological effects of the detected micropollutants in the Baltic Sea. Despite that, a first step was conducted to evaluate the potential risk of occurring micropollutants. For the risk assessment, a risk quotient is calculated as a ratio of the measured environmental concentration and the predicted no-effect concentration (PNEC). For each micropollutant, a risk quotient was calculated by either using a known marine water PNEC or a sensitive freshwater PNEC (Tab. S14) (Ferrari et al. 2004; European Commission 2005c, a, b; Muñoz et al. 2010; Mhadhbi et al. 2012; Ccanccapa et al. 2016; NORMAN Network March 2020).

**Fig. 9:**
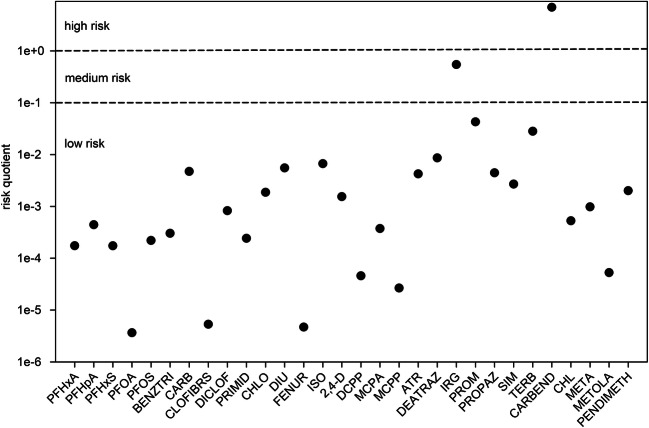
Risk assessment of the median concentrations of the detected micropollutants in the Baltic Sea from 2001 to 2014. Risk quotient < 0.1 low risk; 0.1 ≤ risk quotient ≤ 1 medium risk; 1 ≤ risk quotient high risk. Data: see Tab. S14

Most micropollutants show low risk (risk quotient *<* 0.1) and do not pose acute toxic effects, except for two micropollutants (Fig. 9). IRG could potentially pose a medium risk, whereas for carbendazim (CARBEND), a high risk was calculated for the Baltic Sea. CARBEND was only measured since 2013 at a median concentration of 1.0 ng/L (*n* = 19). Yet, the organism *Daphnia magna* is very sensitive to this fungicide, explaining the high-risk quotient (Ccanccapa et al. 2016). Thus, future research programs in the Baltic Sea should investigate the occurrence and effects of CARBEND.

Of more importance are potential additive and cumulative effects by a set of pollutants and especially possible chronic effects of the pollutant load on aquatic organisms (Magnusson et al. 2010, 2012; Lewis et al. 2012). Effect levels for such chronic effects are presently not available for most micropollutants. Most of the detected concentrations are below environmental assessment criteria, as far as criteria are set at all. However, the detected concentrations within this study are well above background values, which should be zero for a possible chronic effect of the pollutant load anthropogenic substances and above concentrations, which are observed in other open sea areas, such as the open North Sea. Encouraging is that decreasing concentrations of several micropollutants over the investigation decade could be observed and that their single concentrations currently only pose a low risk.

## Conclusion and recommendations

Results demonstrate that there is a multitude of mid-polar organic micropollutants present in the Baltic Sea. In addition to the well-known “classical” pollutants, like PCBsor PAHs, organic micropollutants are currently an unidentified burden to the marine environment. Due to their mostly polar character, they are detectable in the water phase, where their concentrations outrange the classical pollutants by up to three orders of magnitude (Naumann et al. 2020). Nevertheless, the current monitoring programs are lacking observations of the investigated micropollutants, and thus should be updated in the near future.

The spatial distribution of the investigated micropollutants is homogenous throughout the Baltic Sea. A slight decrease in concentration is observed for most compounds from west to east, with the lowest values in the Bothnian Sea. At the station PB1-3, close to the mouth of the Odra, concentrations of some pollutants show high values indicating a riverine input. In summer, locally elevated concentrations of some herbicides were observed at coastal stations in the western Baltic Sea (coast of Schleswig-Holstein), and to a lesser extent in the Gulf of Finland. Additionally, 2,4-D concentration is high at a region north of the Darss and at the Arkona Basin. The observed time trends are quite different for individual micropollutants. Those compounds which exhibit no direct current inputs (e.g., ATR, SIM, PFOA, PFOS) show downward directed time trends, demonstrating the success of the reduction measures taken in the past. Several herbicides (e.g., DIU, 2,4-D) show highly seasonal influences with elevated concentrations during summertime and high local and temporal variabilities. For these compounds, no trend is detectable. Remarkably, for some compounds (e.g., PFHXA, PFHPA, TERB, METOLA), slightly increasing trends can be identified, showing a new negative impact possibly replacing restricted pollutants.

The presented results showed the following gaps: the seasonal coverage was not at an optimum and should be improved. For some compounds (e.g., herbicides), a seasonally influenced input is likely, as a consequence of their application periods. To improve the interpretation, concerning pollution sources, the spatial range of investigation should be enlarged to the entire Baltic Sea, and the layered structure of the Baltic Sea should be represented in the sampling strategy. Knowledge about the distribution of the contaminants could be improved by mathematical modeling, as most of the described polar compounds behave fairly conservative. Additional polar compounds are likely to be present in the Baltic Sea environment. Therefore, screening for new contaminants (e.g., pharmaceuticals, hormones, and antibiotics) should be encouraged, and the monitoring programs should be adapted. A large lack exists concerning the chronic ecological evaluation of the observed concentrations of the micropollutants. Hence, research concerning eco-toxicological data for chronic effects of single compounds or mixtures in the marine environment is needed.

## Supplementary information

ESM 1 (XLSX 5.68 mb)

## Data Availability

All data generated or analyzed during this study are included in this published article and its supplementary information files.
